# Population-Based Incidence Rates of First-Ever Stroke in Central Vietnam

**DOI:** 10.1371/journal.pone.0160665

**Published:** 2016-08-11

**Authors:** Hirotomo Yamanashi, Mai Quang Ngoc, Tran Van Huy, Motoi Suzuki, Akira Tsujino, Michiko Toizumi, Kensuke Takahashi, Vu Dinh Thiem, Dang Duc Anh, Nguyen Thi Hien Anh, Le Huu Tho, Takahiro Maeda, Sharon E. Cox, Lay-Myint Yoshida, Koya Ariyoshi

**Affiliations:** 1 Department of Clinical Medicine, Institute of Tropical Medicine, Nagasaki University Graduate School of Biomedical Sciences, Sakamoto, Nagasaki, Japan; 2 Department of Island and Community Medicine, Nagasaki University Graduate School of Biomedical Sciences, Goto, Nagasaki, Japan; 3 Department of Geriatrics, Khanh Hoa Gerenral Hospital, Nha Trang, Khanh Hoa, Vietnam; 4 Department of Neurology and Strokology, University Graduate School of Biomedical Sciences, Sakamoto, Nagasaki, Japan; 5 Department of Pediatric Infectious Diseases, Institute of Tropical Medicine, Nagasaki University, Nagasaki, Japan; 6 National Institute of Hygiene and Epidemiology, Hanoi, Vietnam; 7 Khanh Hoa Health Service, Nha Trang, Khanh Hoa, Vietnam; 8 Department of Community Medicine, Nagasaki University Graduate School of Biomedical Sciences, Sakamoto, Nagasaki, Japan; 9 Nagasaki University School of Tropical Medicine and Global Health, Sakamoto, Nagasaki, Japan; 10 London School of Hygiene and Tropical Medicine, London, United Kingdom; Massachusetts General Hospital, UNITED STATES

## Abstract

**Introduction:**

Stroke incidence data with methodologically acceptable design in Southeast Asia countries is limited. This study aimed to determine incidence of age-, sex- and subtype-specific first-ever stroke (FES) in Vietnam.

**Methods:**

We conducted a hospital-based retrospective study, targeting all stroke cases hospitalized at a solo-provider hospital in our study site of Nha Trang from January 2009 to December 2011 with International Classification of Diseases, 10th revision (ICD-10) codes I60-69. We calculated positive predictive values (PPVs) of each ICD-10-coded stroke by conducting a detailed case review of 190 randomly selected admissions with ICD-10 codes of I60-I69. These PPVs were then used to estimate annual incident stroke cases from the computerized database. National census data in 2009 was used as a denominator.

**Results:**

2,693 eligible admissions were recorded during the study period. The crude annual incidence rate of total FES was 90.2 per 100,000 population (95% CI 81.1–100.2). The age-adjusted incidence of FES was 115.7 (95% CI 95.9–139.1) when adjusted to the WHO world populations. Importantly, age-adjusted intracerebral hemorrhage was as much as one third of total FES: 36.9 (95% CI 26.1–51.0).

**Conclusions:**

We found a considerable proportion of FES in Vietnam to be attributable to intracerebral hemorrhage, which is as high or exceeding levels seen in high-income countries. A high prevalence of improperly treated hypertension in Vietnam may underlie the high prevalence of intracerebral hemorrhagic stroke in this population.

## Introduction

### Incidence and effects of stroke in low- and middle-income countries

Stroke is the second leading cause of death and third leading cause of loss of disability adjusted life years (DALYs) globally and is estimated to be rapidly increasing in low- and middle-income countries (LMICs) [1–4]. So far, published stroke incidence data with methodologically reliable design is limited in LMICs. The recent studies in LMICs showed relatively higher age-adjusted first-ever stroke (FES) incidence per 100,000 person-years compared to high-income countries; 108.2 in Chile, 116.1 in Georgia, 137 in Brazil, and 135 in India in LMICs versus 97 in Italy, 102.8 in Barbados, 73 in UK, 102.9 in New Zealand [5–13].

### International comparison of stroke incidence

Comparing stroke incidence in different parts of the world is complex and standardized methods are needed [14]. The WHO MONICA study was a landmark study, comparing stroke incidence from participating centers in Europe, Russia, and China [15]. However, this study had several limitations: a) no center included participants over 75 years old; b) it was not designed to investigate incidence by different pathological types; and c) it was not prospective in design. Feigin *et al*., proposed criteria for an ideal study of stroke incidence, which included definitions of FES and recurrent stroke, complete population-based case ascertainment and a prospective study design [16]. Because standard definition of stroke requires imaging test such as brain CT scan or MRI, which are not readily available in LMICs, information of pathological type-specific stroke incidence has mostly been confined to high-income countries [17].

### Stroke studies in Southeast Asia

Stroke is ranked as the number one cause of loss of DALYs in Southeast Asia [1]. However, no study from Southeast Asia has reported stroke incidence which meets Feigin’s standard definitions and methods criteria [16]. Thus reliable data of population-based FES incidence remains unavailable in Southeast Asia.

### Study objectives

We conducted the present study with the objective of estimating the population-based incidence of age-, sex- and subtype-specific FES in Vietnam by utilizing our previously established, large-scale, community-based field site in Nha Trang city, central Vietnam.

## Methods

### Study location

The current study was conducted in Nha Trang, the capital city of Khanh Hoa Province in South-central Vietnam. The city comprises 19 urban and 8 suburban communes, with a total population of 392,279 in 2009. The Khanh Hoa General Hospital (KHGH) is located in the center of Nha Trang and provides primary to tertiary care for residents. The hospital has an 800-bed capacity, being equipped with two CT scanners and one MRI scanner. KHGH served as the sole provider of hospital acute admission care in the city at the time of study. All admissions in KHGH were recorded in a computerized database with International Classification of Diseases, 10th revision (ICD-10) coding along with age, gender, date of admission, discharge status, and address codes.

### Study design

We conducted a hospital-based retrospective study, targeting all stroke cases hospitalized at KHGH from January 2009 through to December 2011 (36 months). Eligible cases were identified from the hospital admission database, based on the criteria of ICD-10 code of I60-69 and residing in Nha Trang. To verify the diagnosis of subtype specific FES, 190 medical charts meeting the ICD-10 code criteria were randomly selected and reviewed by a study clinician and an independent neurologist. A standardized form was used to collect demographic information, history of stroke, external injury, hypertension, diabetes mellitus, neurological signs, stroke subtype diagnosis, performing time and remarks of brain CT/ MRI, and status at discharge.

Stroke was defined according to the WHO clinical definition, as a focal (or at times global) neurological impairment of sudden onset, and lasting more than 24 hours (or leading to death), and of presumed vascular origin [18]. Transient ischemic attack (TIA) was excluded. A stroke case was further classified into one of four subtypes: 1. Ischemic stroke (cases of stroke for which brain CT undertaken on or before the discharge date, showed an area of low attenuation in a region compatible with the clinical picture or for which CT was normal but fulfilled the WHO criteria): 2. Intracerebral hemorrhage (brain CT showed an area of high attenuation in a region compatible with clinical signs and symptoms): 3. Subarachnoid hemorrhage (acute onset of severe headache, sometimes associated with loss of consciousness, seizures, or focal neurological signs not associated with trauma, and a brain CT scan showing subarachnoid or cisternal high attenuation): 4. Undetermined stroke (histories and signs were compatible with stroke but brain CT findings were not available). FES was distinguished from recurrent stroke by no history of previous stroke on medical charts. A case with only a history of TIA was regarded as FES.

### Estimation procedure of first-ever stroke incidence rates

Population-based incidence rates of age- and subtype-specific FES were estimated as follows. Firstly, we calculated positive predictive values (PPVs) of true FES subtype for each ICD-10-coded stroke (I60-69) as a similar approach was applied in previous studies [19–21] and our preceding medical chart review revealed that the discharge diagnosis included some ineligible recurrent strokes and a number of miscoding diagnoses, varying by ICD-10-code. In the current study, PPV was defined as the proportion of confirmed cases of FES subtypes by the study clinician and neurologist where the denominator was the total number of reviewed cases with each of ICD-10-coded stroke (I60-69). We also calculated PPVs of targeted FES subtype for miscoded cases to take the miscoding cases into account.

Secondly, we estimated the annual number of age-, sex-specific FES cases admitted to KHGH. Out of all the discharge summaries in KHGH, which were assigned to an ICD-10 diagnosis, we counted all ICD-10-coded stroke cases during the three year study period and multiplied the number of cases for each ICD-10-code by the PPV of each ICD-10-code for the targeted FES subtype. The annual number of each FES subtype was estimated by summing up all targeted FES subtypes, including miscoded cases classified elsewhere; then divided by three to obtain the annual incidence.

Thirdly, based on the assumption that all FES cases in Nha Trang city were admitted to KHGH, the age-, sex- and subtype-specific FES incidence rates were calculated by dividing the estimated annual incidence by the number of the relevant population in the city in a certain age group. To compare our estimated incidence with other countries, direct age standardizations were performed using two standard populations: the Vietnamese population in 2009 according to the national census [22], and the WHO standard population [23]. The 95% confidence intervals (CI) for the incidence rates were calculated using Poisson regression. Characteristics of first-ever ischemic stroke and intracerebral hemorrhage cases confirmed in medical chart review were compared using chi-square tests for categorical variables and Student’s t-tests for continuous variables. STATA 14.0 was used for all statistical analyses. The Institutional Review Board of the National Institute of Hygiene and Epidemiology, Hanoi, Vietnam, and of the Institute of Tropical Medicine, Nagasaki University, Nagasaki, Japan approved this study (130606111). We did not obtain informed consent because the data was analyzed anonymously.

## Results

### Medical chart review

Medical chart review revealed 75 FES and 19 recurrent stroke cases among the randomly selected 190 case charts with ICD-10 coding of I60-69. Of the FES cases, 38 (50.7%) and 25 (33.3%) cases were ischemic stroke and intracerebral hemorrhage, respectively. There were 2 (2.7%) cases of subarachnoid hemorrhage. 65 (87%) cases had CT/MRI results but 10 cases (13.3%) were categorized as undetermined subtype due to lack of brain CT/MRI findings. The remaining non-stroke cases were ascertained to be due to the following diseases: headache (n = 51), TIA (n = 12), dizziness (n = 10), subdural hematoma (n = 3), pneumonia (n = 3), brain tumor (n = 2), cardiopulmonary arrest (n = 1), epilepsy (n = 1), hypoglycemia (n = 1), alcohol poisoning (n = 1), hypertension (n = 1), syncope (n = 1), traffic accident (n = 1), and undiagnosed (n = 8).

Characteristics of the subtype specific FES cases are summarized in Table 1. The mean age of intracerebral hemorrhage was younger than ischemic stroke, though the difference was not statistically significant (p = 0.141). Nearly 90% of total FES cases and 100% of hemorrhagic stroke cases were directly admitted rather than referred via clinics. The majority of FES cases had past history of hypertension, diabetes mellitus or dyslipidemia, though one third of intracerebral hemorrhage cases were not diagnosed with hypertension prior to this admission. Survival status at discharge was available in all FES cases. Of those, 3 patients died in hospital and 10 (76.9%) patients were discharged in grave condition to die at home, resulting in case fatality rate of 17.3 (95% CI 9.2–29.6). The case fatality rate was significantly higher among the 25 intracerebral hemorrhage cases than 38 ischemic stroke cases (p<0.001).

**Table 1:** Characteristics of cases confirmed as first-ever stroke by pathological subtype in medical chart review. Mean ± standard deviation or n (%) are shown; age, admission period of subarachnoid hemorrhage show individual values.

	All first-ever stroke (N = 75)	Stroke subtype				*p* value (ischemic stroke vs. intracerebral hemorrhage)
Variables		Ischemic stroke (N = 38)	Intracerebral hemorrhage (N = 25)	Subarachnoid hemorrhage (N = 2)	Undetermined (N = 10)	
Age, years	63 ± 13.6	64.8 ± 11.4	60.6 ± 17.1	57, 63	62.9 ± 13	0.141
Men	34 (45.3)	17 (44.7)	13 (52)	2 (100)	2 (20)	0.572
Referred from clinics before admission	8 (10.7)	5 (13.2)	0	0	3 (30)	0.071
Medical insurance	40 (53.3)	21 (55.3)	13 (52)	1 (50)	5 (50)	0.799
Hospital admission fee, USD	195	193	260	121	84	0.174
Individual payment, USD	97	84	143	48	58	0.115
Past medical history						
Hypertension	55 (73.3)	29 (76.3)	17 (68)	0	9 (90)	0.467
Diabetes mellitus	11 (14.7)	8 (21.1)	2 (8)	0	1 (10)	0.165
Dyslipidemia	1 (1.3)	1 (2.6)	0	0	0	0.603
Admission period	8.9 ± 6.5	9 ± 6	8.4 ± 7.8	3, 36	9.8 ± 5	0.427
Fatality	13 (17.3)	2 (5.2)	11 (44)	0	0	<0.001

### Positive Predictive Values of ICD-10-coded stroke

The PPV of each ICD-10-coded stroke subtype was calculated (Table 2). True cerebral hemorrhage cases were recorded mainly as I61 but also some cases were recorded as I63, I64 and I67. Therefore to take the miscoding cases into account, for example, we calculated PPVs of first-ever intracerebral hemorrahge not only over I61-coded stroke (PPV 0.633) but also over miscoded stroke, which were coded as I63 (PPV 0.022), I64 (0.071) and I67 (0.030). True ischemic stroke cases were recorded as I63 (PPV 0.609) and also miscoded as I64 (0.214) and I61 (0.033). True undetermined stroke cases were recorded as I64 (PPV 0.143) and I63 (0.087). The reviewed 190 cases contained two confirmed subarachnoid hemorrhage cases but these were recorded as I61 (PPV 0.033) and I64 (0.024) but not as I60. Thus 0.633, PPV of I61 was assigned to the PPV of I60 since other published studies showed PPVs were similar between I60 and I61 [20, 21].

**Table 2:** Results of medical chart review and positive predictive values. FES indicates first-ever stroke.

		ICD-10 code									
	Total	I60 (Subarachnoid hemorrhage)	I61 (Intracerebral hemorrhage)	I62 (Other nontraumatic intracranial hemorrhage: subdural hemorrhage etc.)	I63 (Cerebral infarction; ischemic stroke)	I64 (Stroke, not specified as hemorrhage or infarction: undetermined stroke)	I65 (Occlusion and stenosis of precerebral arteries)	I66 (Occlusion and stenosis of cerebral arteries)	I67 (Other cerebrovascular diseases: cerebral aneurysm etc.)	I68 (Cerebral amyloid angiopathy)	I69 (Sequelae of cerebrovascular disease)
Reviewed cases (N)	190	**0**	**30**	1	**46**	**42**	1	1	**66**	3	0
**Confirmed diagnosis**											
Ischemic stroke	38	0	**1**	0	**28**	**9**	0	0	**0**	0	0
Intracerebral hemorrhage	25	0	**19**	0	**1**	**3**	0	0	**2**	0	0
Subarachnoid hemorrhage	2	0	**1**	0	**0**	**1**	0	0	**0**	0	0
Undetermined stroke	10	0	**0**	0	**4**	**6**	0	0	**0**	0	0
**Positive Predictive Values**											
Ischemic stroke		-	0.033	-	0.609	0.214	-	-	-	-	-
Intracerebral hemorrhage		-	0.633	-	0.022	0.071	-	-	0.030	-	-
Subarachnoid hemorrhage		0.633*	0.033	-	-	0.024	-	-	-	-	-
Undetermined stroke		-	-	-	0.087	0.143	-	-	-	-	-

* The PPV of I61 (0.633) was assigned.

### Estimated annual number of first-ever stroke cases

According to the ICD-10-code database, a total of 4,344 stroke cases were admitted to KHGH during the 3 years of study period. Among them, 2,693 (62%) were residents of Nha Trang thus eligible for the current analysis. The age-, sex- and subtype-specific distribution of ICD-10-coded stroke cases admitted to KHGH are shown in S1 Table. 1,287 (47.8%) were males and the mean age was 60.1 (Standard Deviation, SD 18.0) years. Discharage status was missing in one case. In total 258 (9.5%) died or were discharged in critical condition but this ranged from 0% to 34.4% depending on ICD-10 code: 0/7 (0%) in subarachnoid hemorrhage (ICD-10-I60), 148/430 (34.4%) Intracerebral hemorrhage (ICD-10-I61), 50/702 (7.1%) ischemic stroke (ICD-10-I63), 57/503 (11.3%) undetermined (ICD-10-I64), and 1/887 (0.1%) other cerebral vascular diseases (ICD-10-I67). Mean duration of admission among those who survived was 9.9 days (SD 9.6) in all cases, ranging from 0 to 116 days: 11.3 (SD 13.7) for ICD-10-I60, 11.5 (SD 11.0) for ICD-10-I61, 11.5 (SD 10.1) for ICD-10-I63, 10.4 (SD 9.8) for ICD-10-I64, and 7.6 (SD 8.2) for ICD-10-I67.

Estimated number of annual FES cases for each subtype were calculated by multiplying the total number of age-, sex- and subtype-specific ICD-10-coded cases by the PPV then divided by three years (S2 Table). For example, the total number of intracerebral hemorrhage cases for three years was estimated as the sum of I61-coded stroke cases multiplied by the PPV of I61 to predict intracerebral hemorrhage, the number of I63-coded stroke cases multiplied by PPV of I63 to predict intracerebral hemorrhage (miscoding no.1), the number of I64-coded stroke cases multiplied by PPV of I64 to predict intracerebral hemorrhage (miscoding no.2), and the number of I67-coded stroke cases multiplied by PPV of I67 to predict intracerebral hemorrhage (miscoding no.3). Similarly, to estimate total number of ischemic stroke cases, the numbers of cases categorized as I61, I63 and I64 were multiplied by each codes PPVs for ischemic stroke and summed; to estimate total number of undetermined stroke cases, the numbers of cases categorized as I63 and I64 were multiplied by each codes PPVs for undetermined stroke and summed. For estimation of the total number of subarachnoid hemorrhages, the numbers of cases categorized as I60, I61 (miscoded cases) and I64 (miscoded cases) were multiplied by each PPVs and summed.

### Stroke incidence rates

The crude annual incidence rate of total FES was 90.2 per 100,000 population (95% CI 81.1–100.2) (Table 3). When adjusted to the WHO world populations or Vietnamese population of 2009, the age-adjusted incidence of FES was 115.7 (95% CI 95.9–139.1) and 95.7 (77.8–117.2), respectively. The total stroke incidence increased with age with the peak of 1055.7 in people aged 85 years or over. This increasing tendency with age was not seen in intracerebral hemorrhage incidence in men between aged 75–84 and aged 85 years or over, as well as ischemic stroke in women and total FES. Crude incidence in the oldest age group women (aged ≥85 yrs) exceeded that of men.

**Table 3:** Age-, sex-specific and age-adjusted incidence rates per 100,000 of first-ever stroke, all and by pathological subtype.

	Total population (N)	Ischemic stroke		Intracerebral hemorrhage		Subarachnoid hemorrhage		Undetermined stroke subtype		Total stroke	
		Estimated number of cases	Incidence rate (95% CI)	Estimated number of cases	Incidence rate (95% CI)	Estimated number of cases	Incidence rate (95% CI)	Estimated number of cases	Incidence rate (95% CI)	Estimated number of cases	Incidence rate (95% CI)
Male											
Age group, years											
<15	46,319	0.1	0	0.7	2.2 (0.1–12)	0.0	0	0.0	0	0.9	2 (0.1–12)
15–24	37,574	0.2	0	0.8	2.7 (0.1–14.8)	0.2	0	0.0	0	1.3	2.7 (0.1–14.8)
25–34	32,911	0.1	0	0.9	3.0 (0.1–16.9)	0.0	0	0.0	0	1.1	3.0 (0.1–16.9)
35–44	32,715	5.2	15.3 (5–35.7)	11.3	33.6 (16.8–60.2)	0.8	3.1 (0.1–17)	1.1	3.1 (0.1–17)	18.4	55.0 (32.6–87)
45–54	22,081	18.7	86.1 (51.8–134.4)	16.0	72.5 (41.4–117.7)	1.6	9.1 (1.1–32.7)	5.0	22.6 (7.4–52.8)	41.3	185.7 (133.3–251.9)
55–64	9,136	21.3	229.9 (142.3–351.4)	15.3	164.2 (91.9–270.8)	1.6	21.9 (2.7–79.1)	5.0	54.7 (17.8–127.7)	43.2	470.7 (340.6–634)
65–74	4,910	23.8	488.8 (313.2–727.3)	12.2	244.4 (126.3–426.9)	0.9	20.4 (0.5–113.5)	5.1	101.8 (33.1–237.6)	42.1	855.4 (616.5–1156.3)
75–84	3,373	23.5	711.5 (455.9–1058.7)	10.4	296.5 (142.2–545.2)	0.8	29.7 (0.8–165.2)	5.2	148.2 (48.1–345.9)	40.0	1185.9 (847.2–1614.8)
≥85	718	6.2	835.7 (306.7–1818.9)	2.2	278.6 (33.7–1006.2)	0.3	0	1.8	278.6 (33.7–1006.2)	10.5	1532.0 (764.8–2741.2)
Total	1,89,737	99.2	52.2 (42.4–63.5)	69.8	36.9 (28.8–46.6)	6.4	3.2 (1.2–6.9)	23.4	12.1 (7.7–18.2)	198.7	104.9 (90.8–120.5)
Female											
Age group, years											
<15	42,441	0.2	0	0.0	0	0.0	0	0.0	0	0.2	0
15–24	42,400	0.4	0	1.4	2.4 (0.1–13.1)	0.0	0	0.1	0	1.9	4.7 (0.6–17)
25–34	35,128	1.0	2.9 (0.1–15.9)	1.3	2.9 (0.1–15.9)	0.0	0	0.3	0	2.7	8.5 (1.8–25)
35–44	33,013	3.5	9.1 (1.9–26.6)	3.5	12.1 (3.3–31)	0.2	0	1.0	3.0 (0.1–16.9)	8.1	24.2 (10.5–47.8)
45–54	23,833	7.5	33.6 (14.5–66.1)	7.1	29.4 (11.8–60.5)	0.5	0	2.0	8.4 (1–30.3)	17.1	71.3 (41.6–114.2)
55–64	11,773	16.1	135.9 (77.7–220.7)	9.7	84.9 (40.7–156.2)	0.8	8.5 (0.2–47.3)	4.1	34.0 (9.3–87)	30.7	263.3 (178.9–373.8)
65–74	7,871	22.4	279.5 (175.2–423.2)	9.8	127.1 (60.9–233.7)	0.8	12.7 (0.3–70.8)	5.2	63.5 (20.6–148.2)	38.3	482.8 (341.7–662.7)
75–84	4,717	26.8	572.4 (377.2–832.8)	10.3	212.0 (101.7–389.9)	1.2	21.2 (0.5–118.1)	6.7	148.4 (59.7–305.8)	44.9	954.0 (695.9–1276.5)
≥85	1,366	6.0	439.2 (161.2–956)	3.7	292.8 (79.8–749.8)	0.3	0	1.7	146.4 (17.7–528.9)	11.7	878.5 (453.9–1534.5)
Total	2,02,542	84.0	41.5 (33.1–51.4)	47.0	23.2 (17.1–30.9)	3.9	2.0 (0.5–5.1)	20.9	10.4 (6.4–15.9)	155.7	77.0 (65.4–90.1)
Male and Female											
Age group, years											
<15	88,760	0.3	0	0.7	1.1 (0–6.3)	0.0	0	0.1	0	1.1	1 (0–6.3)
15–24	79,974	0.7	1.3 (0–7)	2.2	2.5 (0.3–9)	0.3	0	0.1	0	3.2	3.8 (0.8–11)
25–34	68,039	1.2	1.5 (0–8.2)	2.2	2.9 (0.4–10.6)	0.1	0	0.3	0	3.8	5.9 (1.6–15.1)
35–44	65,728	8.6	13.7 (6.3–26)	14.8	22.8 (12.8–37.6)	1.0	1.5 (0–8.5)	2.0	3.0 (0.4–11)	26.5	41.1 (27.1–59.8)
45–54	45,914	26.2	56.6 (37–83)	23.2	50.1 (31.8–75.2)	2.1	4.4 (0.5–15.7)	6.9	15.3 (6.1–31.4)	58.4	126.3 (95.9–163.3)
55–64	20,909	37.4	177.0 (124.6–243.9)	25.1	119.6 (77.4–176.5)	2.3	9.6 (1.2–34.6)	9.1	43.0 (19.7–81.7)	73.9	353.9 (277.9–444.3)
65–74	12,781	46.3	359.9 (263.5–480.1)	22.0	172.1 (107.9–260.6)	1.7	15.7 (1.9–56.5)	10.4	78.2 (37.5–143.9)	80.4	625.9 (496.3–779)
75–84	8,090	50.3	618.1 (458.7–814.8)	20.7	259.6 (160.7–396.8)	2.0	24.7 (3–89.3)	11.9	148.3 (76.6–259.1)	84.9	1050.7 (839.3–1299.2)
≥85	2,084	12.2	575.8 (297.5–1005.8)	6.0	287.9 (105.7–626.7)	0.6	48.0 (1.2–267.4)	3.5	191.9 (52.3–491.4)	22.3	1055.7 (661.6–1598.3)
Total	3,92,279	183.1	46.7 (40.1–53.9)	116.8	29.8 (24.7–35.8)	10.2	2.6 (1.2–4.7)	44.3	11.2 (8.2–15.1)	354.5	90.2 (81.1–100.2)
Age-adjusted by Vietnam*			49.6 (37.1–65.9)		31.1 (21.1–44)		2.7 (0.6–8.8)		12.1 (6.2–21)		95.7 (77.8–117.2)
Age-adjusted by WHO world**			60.7 (46.7–78.4)		36.9 (26.1–51)		3.2 (0.6–8.8)		14.6 (8.4–24.7)		115.7 (95.9–139.1)

*2009 Vietnam national census.

**WHO world standard.

Ischemic stroke was the most common subtype but it is noteworthy that intracerebral hemorrhage constituted as much as one third of total stroke in this population. When the subtype-specific FES incidence rates were calculated, the crude incidence of ischemic stroke was only 1.57 fold greater than hemorrhagic: 46.7 (95% CI 40.1–53.9) vs 29.8 (95% CI 24.7–35.8), respectively. The incidence of FES was higher in men than in women regardless of pathological type. However total number of women admitted to KHGH with ICD-10-coded stroke was higher than men. This discrepancy was mainly attributed to the considerably higher number of women admitted with ICD-10-I67 (S1 Table). Subarachnoid hemorrhage was the least common in FES contributing only 2.9% to the incidence rate. The fatality rate of subarachnoid hemorrhage was quite low in both chart review and ICD-10-coded stroke cases (I60).

## Discussion

This is the first population-based study in southeast Asia, documenting age-, sex- and subtype-specific stroke incidence rates. We found a considerable proportion of FES in central Vietnam to be attribute to intracerebral hemorrhage and age-adjusted incidence rate of intracerebral hemorrhage at 36.9 is the highest reported since 2000, including other LMICs (Table 4) [5–13]. It is plausible that this observation may be due to substantial numbers of hypertensive patients not being either diagnosed, treated or suboptimaly treated in Vietnam.

**Table 4:** Age-adjusted stroke incidence and proportion of pathological type, by high-income or low- and middle-income countries. NR = not reported. The direct age standardizations were performed using WHO world standard populations.

Study site	Study duration (year)	Age-adjusted incidence of first-ever stroke per year/100,000 population (95% CI)	Age-adjusted incidence of ischemic stroke per year/100,000 population (95% CI)	Age-adjusted incidence of intracerebral hemorrhage per year/100,000 population (95% CI)	Age-adjusted incidence of subarachnoid hemorrhage per year/100,000 population (95% CI)	Age-adjusted incidence of undetermined stroke per year/100,000 population (95% CI)
***High-income countries***						
Valley d' Aosta, Italy^(5)^	2004–2005	97 (80–114)	75 (60–90)	10 (5–15)	5 (1–9)	8 (3–13)
Barbados, Barbados ^(6)^	2001–2002	102.8 (84.1–124.9)†	88.3 (70.6–108.4)†	13.3 (6.9–22.2)†	2.8 (0.6–8.8)†	3.6 (1.1–10.2)†
Oxfordshire, UK^(7)^	2002–2004	73 (64–83)	NR	NR	NR	NR
Auckland, New Zealand^(8,9)^	2002–2003	102.9 (84.1–124.9)¶	75.2 (59–94)¶	13.2 (6.9–22.2)¶	6.9 (2.8–14.4)¶	7.6 (3.5–15.8)¶
***Low- and middle-income countries***						
Iquique, Chile^(10)^	2000–2002	108.2 (95.8–120.6)	63.9 (49.3–81.7)†	22.2 (13.8–33.3)†	NR	NR
Tbilisi, Georgia^(11)^	2000–2003	116.1 (95.9–139.1)†	60.3 (45.8–77.2)†	29.3 (19.4–41.7)†	14.1 (7.7–23.5)†	12.5 (6.9–22.2)†
Matao, Brazil^(12)^	2003–2004	137 (112–166.4)	122.9 (102.2–146.8)†	20.0 (13–32.1)†	NR	NR
Trivandrum, India^(13)^	2005	135 (123–146)	74.8 (66.3–83.2)	10.1 (7–13.2)	4.2 (2.2–6.1)	NR
Nha Trang, Vietnam	2009–2011	115.7 (95.9–139.1)	60.7 (46.7–78.4)	36.9 (26.1–51)	3.2 (0.6–8.8)	3.2 (0.6–8.8)

† Calcurated by the author, based on the reports.

¶ Calcurated by the author, based on the reports. Incidence rate aged <15 assumed zero.

It has been reported that the incidence of hemorrhagic stroke is more strongly associated with blood pressure than ischemic stroke [24, 25]. A similar situation existed in Japan about 50 years ago where the incidence and mortality of intracerebral hemorrhage was very high particularly among men with untreated or sub-treated hypertension [26]. A previous community-based studies in Vietnam in 2002–2008 reported only 17.4–48.4% of hypertensive patients were aware of their blood pressure status, and only 29.6–36.7% of the hypertension patients who were aware of their condition, were under treatment with anti-hypertensive drugs [27, 28]. National representative verbal autopsy survey in 2006–2007 in Vietnam found that cerebrovascular diseases and hypertensive diseases were ranked within top three in both sexes [29]. Following a lesson learned in Japan that the incidence of intracerebral hemorrhage in Japanese men steeply declined from 1961 to 2002 along with the dissemination of antihypertensive drug use from 2.0% to 17.5% in the study population [26], community-based blood pressure screening and treatment dissemination should be urgenly implimented in Vietnam.

### Projection of stroke patients in Vietnam in 2010 and 2030

We predict a dramatic increase in the number of stroke cases as the Vietnamese population ages. Life expectancy at birth increased rapidly from 6.1 to 8.4 years for men, and from 7.3 to 8.9 years in women for two decades in Vietnam, Lao, and Cambodia [30]. According to national census 2009, the population pyramid of Vietnam was bell-shaped, which is compatible with that of our study population in Nha Trang [22]. Six percent of the Vietnamese population is older than 65 years old in 2009. We estimated the total number of FES patients in Vietnam in 2010 and 2030, assuming that age- and sex-specific stroke incidence rates do not vary across the country or over time and using the Vietnamese population in 2010 and predicted population in 2030 [22]. The total number of male and female patients in each age group was calculated by multiplying the predicted age- and sex-specific population by the age- and sex-specific crude incidence rates of our current study. We found the total number of FES cases in Vietnam will increase 1.85 fold (83,546 in 2010 to 154,777 in 2030) by the year 2030.

e estimated the predicted o f fr of stroke patients in 2030 i PPV of I60 to predict subarachnoid hemorrhage because PPVs to f f

e estimated the predicted o f fr of stroke patients in 2030 i PPV of I60 to predict subarachnoid hemorrhage because PPVs to f f

### Limitations

Potential limitations of this study include the possible underestimation of the true FES incidence due to ascertainment from a single hospital (and not including private clinics) and limited to those with hospital ICD-10-coded stroke which could result in missed cases. However, in this study setting, there was no other hospital with admission facility for stroke patients, and also we assumed that economic barriers to hospital admission was low in Vietnam, because it used to be a socialist country and the majority of residents hold health insurance [31]. On the other hand, observed low fatality rate of patients with subarachnoid hemorrhage may indicate that our study missed some pre-hospital death cases. Furthermore our study design was retrospective. Nevertheless, these limitations don’t alter our conclusion of a high age-adjusted FES and intracerebral hemorrhage incidence in comparison with other countries. Finally, our study doesn’t consider possible changes of denominator during the study period of 2009–2011 due to a single population census in 2009.

## Conclusions

The age-adjusted incidence rate of FES in central Vietnam is compatible with previous reports showing stroke incidence rates in LMICs as high or exceeding levels seen in high-income countries. Our findings also highlight the relatively high proportion of intracerebral hemorrhage, which is important to consider screening and preventive strategies in Vietnam.

## Supporting Information

S1 Table Total number of age-, sex-specific ICD-10-coded stroke cases admitted to Khanh Hoa General Hospital who were residing in Nha Trang. (PDF)

S2 Table Estimated annual number of age-, sex-specific true first-ever stroke cases admitted to Khanh Hoa General Hospital by stroke subtypes. (PDF)

## Abbreviations

DALYs: disability adjusted life years
LMICs: low- and middle- income countries
FES: first-ever stroke
KHGH: Khanh Hoa General Hospital
ICD-10: International Classification of Diseases, 10th revision
TIA: transient ischemic attack
PPVs: positive predictive values
CI: confidence intervals
SD: standard Deviation
