# Supplementary material for: Population-Based Incidence Rates of First-Ever Stroke in Central Vietnam
Source: PLoS One. 2016 Aug 11;11(8):e0160665. doi: 10.1371/journal.pone.0160665 (PMC4981455; doi:10.1371/journal.pone.0160665)
Supplement: S1 Table — (PDF) [file pone.0160665.s001.pdf]

Age group, years	ICD-10 code										I60-69
	I60 (Subarachnoid hemorrhage)	I61 (Intracerebral hemorrhage)	I62 (Other nontraumatic intracranial hemorrhage: subdural hemorrhage etc.)	I63 (Cerebral infarction; ischemic stroke)	I64 (Stroke, not specified as hemorrhage or infarction: undetermined stroke)	I65 (Occlusion and stenosis of precerebral arteries)	I66 (Occlusion and stenosis of cerebral arteries)	I67 (Other cerebrovascular diseases: cerebral aneurysm etc.)	I68 (Cerebral amyloid angiopathy)	I69 (Sequelae of cerebrovascular disease)	
Male											
<15	0	3	0	0	1	0	0	1	0	0	5
15-24	1	2	0	1	0	0	0	37	1	1	43
25-34	0	3	0	0	1	0	1	25	0	0	30
35-44	1	49	0	19	11	0	1	53	0	0	134
45-54	2	64	1	66	64	0	5	51	2	2	257
55-64	2	62	2	82	56	0	7	30	0	1	242
65-74	0	47	2	98	48	0	16	41	3	1	256
75-84	0	38	1	96	51	0	10	45	3	0	244
≥85	0	6	0	21	26	0	4	19	0	0	76
Total	6	274	6	383	258	0	44	302	9	5	1287
Female											
<15	0	0	0	1	0	0	0	1	0	0	2
15-24	0	3	0	2	0	0	1	75	0	0	81
25-34	0	2	0	4	3	0	2	77	0	0	88
35-44	0	9	0	12	13	0	2	120	0	1	157
45-54	0	25	3	27	25	0	7	105	2	2	196
55-64	0	35	0	60	49	0	17	75	7	1	244
65-74	0	34	1	89	56	2	18	67	8	1	276
75-84	1	34	1	103	77	0	12	54	6	3	291
≥85	0	14	0	21	22	0	1	11	2	0	71
Total	1	156	5	319	245	2	60	585	25	8	1406
Male and Female											
<15	0	3	0	1	1	0	0	2	0	0	7
15-24	1	5	0	3	0	0	1	112	1	1	124
25-34	0	5	0	4	4	0	3	102	0	0	118
35-44	1	58	0	31	24	0	3	173	0	1	291
45-54	2	89	4	93	89	0	12	156	4	4	453
55-64	2	97	2	142	105	0	24	105	7	2	486
65-74	0	81	3	187	104	2	34	108	11	2	532
75-84	1	72	2	199	128	0	22	99	9	3	535
≥85	0	20	0	42	48	0	5	30	2	0	147
Total	7	430	11	702	503	2	104	887	34	13	2693
