# Supplementary material for: Population-Based Incidence Rates of First-Ever Stroke in Central Vietnam
Source: PLoS One. 2016 Aug 11;11(8):e0160665. doi: 10.1371/journal.pone.0160665 (PMC4981455; doi:10.1371/journal.pone.0160665)
Supplement: S2 Table — (PDF) [file pone.0160665.s002.pdf]

Age group, years	Ischemic stroke	Intracerebral hemorrhage	Subarachnoid hemorrhage	Undetermined stroke	Total
Male					
<15	0.1	0.7	0.0	0.0	0.9
15-24	0.2	0.8	0.2	0.0	1.3
25-34	0.1	0.9	0.0	0.0	1.1
35-44	5.2	11.3	0.8	1.1	18.4
45-54	18.7	16.0	1.6	5.0	41.3
55-64	21.3	15.3	1.6	5.0	43.2
65-74	23.8	12.2	0.9	5.1	42.1
75-84	23.5	10.4	0.8	5.2	40.0
≥85	6.2	2.2	0.3	1.8	10.5
Total	99.2	69.8	6.4	23.4	198.7
Female					
<15	0.2	0.0	0.0	0.0	0.2
15-24	0.4	1.4	0.0	0.1	1.9
25-34	1.0	1.3	0.0	0.3	2.7
35-44	3.5	3.5	0.2	1.0	8.1
45-54	7.5	7.1	0.5	2.0	17.1
55-64	16.1	9.7	0.8	4.1	30.7
65-74	22.4	9.8	0.8	5.2	38.3
75-84	26.8	10.3	1.2	6.7	44.9
≥85	6.0	3.7	0.3	1.7	11.7
Total	84.0	47.0	3.9	20.9	155.7
Male and Female					
<15	0.3	0.7	0.0	0.1	1.1
15-24	0.7	2.2	0.3	0.1	3.2
25-34	1.2	2.2	0.1	0.3	3.8
35-44	8.6	14.8	1.0	2.0	26.5
45-54	26.2	23.2	2.1	6.9	58.4
55-64	37.4	25.1	2.3	9.1	73.9
65-74	46.3	22.0	1.7	10.4	80.4
75-84	50.3	20.7	2.0	11.9	84.9
≥85	12.2	6.0	0.6	3.5	22.3
Total	183.1	116.8	10.2	44.3	354.5
